# Supplementary material for: ShSPI Inhibits Thrombosis Formation and Ischemic Stroke In Vivo
Source: Int J Mol Sci. 2024 Aug 19;25(16):9003. doi: 10.3390/ijms25169003 (PMC11354536; doi:10.3390/ijms25169003)
Supplement: Supplementary file 1 [file ijms-25-09003-s001.zip › ijms-3112639-supplementary.pdf]

Supplementary Information

Supplementary figures and figure legends

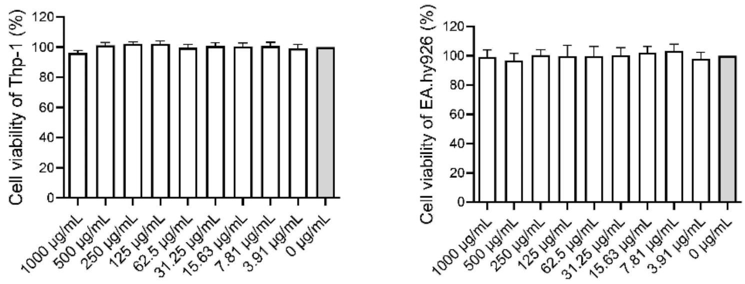

Figure S1. ShSPI showed no cytotoxicity. Cytotoxicity of ShSPI on Thp-1 (A) and EA.hy926 (B) cell was investigated with the concentration from 3.91 to 1000 μg/mL.

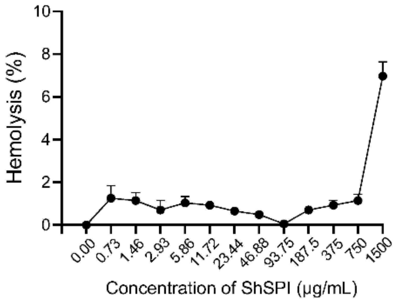

Figure S2. Hemolytic activity of ShSPI. ShSPI only caused about 7% hemolytic activity with its concentration up to 1500 μg/mL, and it showed no influence on the hemolytic activity with the concentration from 0.73 to 750 μg/mL.

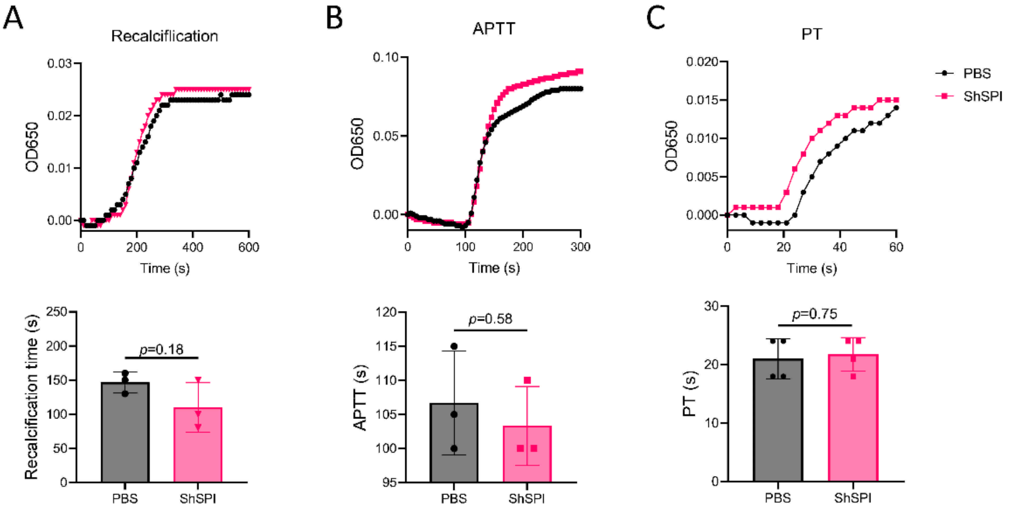

Figure S3. ShSPI showed no direct effects on coagulation cascade. ShSPI showed no effects on the recalcification time (A), APTT (B) and PT (C) at the concentration of 50 μg/mL. Data are mean ± SD.

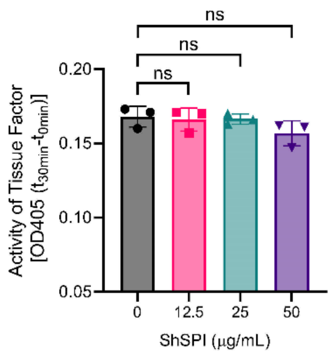

Figure S4. ShSPI showed no effect on tissue factor activity. The activity of tissue factor stimulated with ShSPI (0-50 μg/mL) was assayed using the Tissue Factor Activity Assay Kit according to the instructions.
